# Dichotomic Decision Optimization for the Design of HVDC Superconducting Links

**DOI:** 10.3390/e22121413

**Published:** 2020-12-15

**Authors:** Javier Muñoz-Antón, Adela Marian, Frédéric Lesur, Christian-Eric Bruzek

**Affiliations:** 1 Escuela Técnica Superior de Ingenieros Industriales, Universidad Politécnica de Madrid, 28006 Madrid, Spain; 2 Institute for Advanced Sustainability Studies, 14467 Potsdam, Germany; adela.marian@iass-potsdam.de; 3 Nexans, 62100 Calais, France; frederic.lesur@nexans.com; 4 Nexans, 62301 Lens, France; christian_eric.bruzek@nexans.com

**Keywords:** optimization, bulk power transmission, HVDC superconducting links, MgB_2_, dichotomic decision

## Abstract

Superconducting links are an innovative solution for bulk power transmission, distinguished by their compact dimensions, high efficiency and small environmental footprint. As with any new technology field, there is a large amount of design possibilities for such links, each of them having a profound impact on the system configuration. For instance, changing the material can imply a change in the working temperature from 20 to 70 K and has consequences on the maximum link length. This article presents the dichotomic decision possibilities for the optimized design of a high-power superconducting link, focusing on some of the key components of the cable system. The complex design optimization process is exemplified using the European project Best Paths, in which the first 3-gigawatt-class superconducting cable system was designed, optimized, manufactured, and successfully tested.

## 1. Introduction

Superconductivity occurs in materials that can transmit electricity without losses below three characteristic thresholds: a critical value of the temperature, a critical intensity of the magnetic field, and a critical current density. Discovered more than 100 years ago, superconductivity has found successful application in both research and industrial contexts, for instance, in particle accelerators and medical imaging devices [1]. However, its first envisaged application of perfect electric power transmission has remained untapped for decades, due to the low operating temperatures of the commercially available superconductors. The discovery of high-temperature superconductors in 1987 [2] ushered in new prospects for superconducting power applications, focused in particular on fault current limiters, energy storage, and superconducting cables for power grids [3].

Due to their high efficiency, compact size, and reduced environmental footprint, superconducting power cables have generated increased interest in recent years [4]. These potential advantages are becoming quite relevant, as most countries across the globe are upgrading and expanding their electricity grids as part of their transition to renewable energy.

In the past decade, a growing number of projects related to superconducting cables of various lengths and capacities have been constructed or become operational worldwide [5]. A prominent example of a superconducting cable installed in the grid is the AmpaCity project in downtown Essen, Germany [6]. The 1 km long AC cable is based on high-temperature ceramic superconducting materials and is cooled using liquid nitrogen, operating under a voltage of 10 kV and a current of 2.4 kA. The cable system energizes a full district close to the city center and has been in operation since 2014 with 100% availability. In general, the installation of such superconducting links in inner-city areas would free up valuable space and can lead to greater efficiency and lower operating costs. 

Nevertheless, widespread use of such superconducting systems for power transmission has been hindered by the high costs of the ceramic-based superconductors and their complex manufacturing process. A promising alternative explored in recent years is represented by the simple binary compound magnesium diboride (MgB_2_) [7], which is based on inexpensive raw materials that are abundant in nature. The manufacturing process is much simpler and has already been optimized at industrial level, resulting in commercial availability of MgB_2_ wires in kilometric lengths [8]. Unlike ceramic superconductors, which are cooled by low-cost readily available liquid nitrogen, the cooling medium and the cooling system required by MgB_2_ are more complex and more expensive. The benefits and drawbacks of these two key components of the cable system, superconducting material, and cooling system need to be carefully weighed when designing a new cable and optimizing the system performance.

The optimization process in the design of complex systems is not simply a continuous mathematical function, but goes through a dichotomic decision tree that has not yet been reported for the field of superconducting links. In other engineering fields, there is a multitude of examples of dichotomic decisions related to the design of complex systems:

- Road vehicle traction system [9]: three options are common: front-, rear-, or all-wheel drive. Depending on the chosen use, the weather conditions (snow), the required power, etc., the optimum decision is going to change, affecting the remaining design parameters of the car.
- Airplane engines location [10]: several options are available, for instance, front wings or rear wings. Each of them gives different possibilities in terms of manageability and puts the airplane under different mechanical stresses: compression (rear wings) and traction (front wings).
- Thermal cycle cold sink in power plants [11]: a dry cooling system is cheaper but decreases the cycle efficiency when compared to a wet cooling system, which usually conditions the location of the power plant.
- Windmill morphology [12]: the three-bladed windmill is not the most efficient, but is commonly used at present, due to the balance between cost, reliability, and efficiency.

This work analyses the design possibilities that usually appear in a new technology field—in this case high-power superconducting links—and that can be limited by optimization within the traditional framework of continuous variable analysis. To avoid limitations and achieve an optimal design, dichotomic decisions are introduced, which can bring about a substantial change in the system configuration. We exemplify this approach through an in-depth description of the dichotomic decision-making process undertaken for the recently finished European project Best Paths [13], which demonstrated the first 3-gigawatt-class direct-current superconducting cable system [14,15].

The article is divided into two main sections. Section 2 provides a comprehensive overview of the selection criteria related to available technologies for bulk power transmission, in order to understand their benefits and limitations and be able to situate superconducting links in this context. It also includes a brief introduction to the main components of a superconducting cable system. Section 3 investigates the dichotomic decision possibilities for an optimal design of high-power superconducting links, driven by the Best Paths case study. The focus is set on dichotomic decisions related to the superconductor, cooling fluid, cryogenic envelope, thermal shield, and high-voltage electrical insulation, which affect the design, dimensions, and cost of the cable system.

## 2. Background: Technologies for Bulk Power Transmission

### 2.1. Selection of Link Technology

Available technologies for high-voltage direct-current (HVDC) transmission up-to-day include overhead lines (OHL, bare conductors suspended in air between towers), insulated cables—underground (UGC), or submarine (SUB)—gas insulated lines (GIL, rigid metallic tubes filled with an insulating gas), and superconducting cables (SCC), located underground.

OHL are the backbone of transmission grids, with more than one century of service experience. Generally, except in dense urban states like Singapore, buried technologies represent less than 10% of the installed length, but most of the new infrastructures. GIL and SCC are still niche projects.

OHL are well-established at very high ratings for very long distances with several commissioned and operational ±800 kV links, particularly in China, Brazil, and India. For instance, the Zhundong-Wannan link in China transmits up to 12 GW at ±1100 kV across 3320 km [16]. The longest SUB link has been operating since 2008 over a length of 580 km and a capacity of 700 MW, with the deepest point at 410 m [17]. MurrayLink in Australia is the longest UGC, in operation since 2004, with a length of 177 km and a capacity of 220 MW [18]. The most significant GIL system was commissioned in October 2019 and is the Sudong Tunnel crossing the Yangtze River in China, with a length of 5.4 km and a capacity of 6 GW. This is an AC system, but major developments have recently been achieved in the design and testing of HVDC systems for 5000 A and ±550 kV [19].

**Figure 1:**
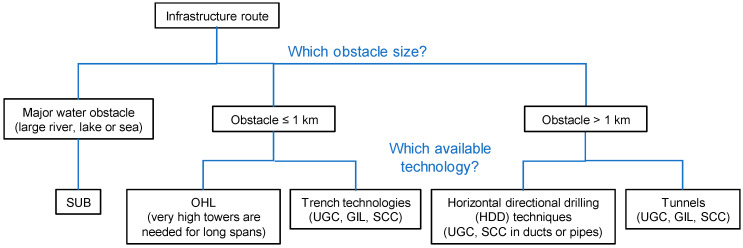
Selection of link technology according to the main obstacle that needs to be crossed.

First of all, the technology selection for new infrastructure depends on the macro-environment to cross, especially according to the nature and size of the obstacles to cross. Common options are highlighted in Figure 1, with the caveat that a few very specific infrastructures push these limits.

### 2.2. Additional Selection Criteria

In addition to the criterion related to obstacles, Table 1 summarizes a number of other important selection criteria for the HVDC transmission technology. Each criterion is rated with stars (up to ★★★★ for high interest) or discs (up to 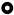

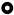

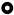

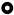
 for high difficulty), depending on whether advantages or disadvantages are described. Furthermore, these various aspects are addressed in detail below, providing a state-of-the-art overview that highlights the complexity of the decision-making process.

#### 2.2.1. Visual Impact

The visual impact of transmission infrastructures may raise strong local opposition. This mainly concerns OHL, as the towers of the corridor become taller and wider with increasing transmission voltage. Buried technologies are often a condition for much better public acceptance and can significantly reduce the duration for getting the necessary administrative permits (typically 3, instead of 10 years).

Most of the existing GIL are located outdoors, but the conduits can be buried or installed in tunnels. SCC are generally installed in buried ducts, with only the terminations visible at the end of the link.

#### 2.2.2. Infrastructure Footprint and Right-of-Way

Insulated solutions are designed to contain the electric fields and are much more compact. However, neither buildings nor trees can be added above the buried cable route.

The required strip of land includes not only the footprint of the power link itself, but also a service strip for eventual maintenance and repair. In any case, the expected footprint during the civil engineering must be extended for temporary storage of the excavated material (typical total width = 50 m, for 16 m trenches hosting 4 parallel circuits in large UGC European projects).

#### 2.2.3. Sensitivity to Climatic Hazards

OHL may be damaged by storms, icing, sticky snow. In areas where these weather conditions are identified and recurrent, it can be preferrable to employ buried technologies. Only the equipment located above the ground in substations would then be affected. Insulation coordination is organised to avoid lightning damages in all power grid sections, as well.

SCC systems can be considered like any regular underground technology.

#### 2.2.4. Sensitivity to Ambient Temperature

The current rating of a resistive transmission link always depends on at least one thermal criterion, because of the Joule losses, which heat the conductor and contribute to its expansion. Moreover, a maximum critical temperature at the outer surface may be required for buried systems, to avoid soil drying and thermal runaway.

The bare conductors of OHL are exposed to solar radiation and cooled by natural convection and wind. The dilatation of a span between two towers brings the lowest point closer to the ground. The regulations impose a safe distance for people.

The cables insulated with impregnated paper or polymeric materials must not overheat beyond a maximum temperature, above which ageing and breakdown may occur.

The intrinsic cryogenic cooling of superconductors makes the SCC independent from the surrounding thermal environment. The current rating is not affected by high ambient temperatures, and no heating is generated to the surrounding soil.

#### 2.2.5. Sensitivity to Rocky Environment

The crossing of mountains is technologically easier with OHL, but results in a strong impact on the surroundings. Tracks are built to access the tower locations; helicopters can be used to carry steel angles. Trenching may be complex in soils with rocks or slopes. Tunnels may be an expensive solution.

When SUB cannot be buried, rock damping techniques ensure their protection.

For SCC, the management of elevation must also be considered due to the pumping of cryogenic fluid.

#### 2.2.6. Ease of Making Joints

Mid-span tension joints provide electrical and mechanical continuity of the OHL conductor. Insulated cables are connected by joints that reproduce the continuity of each of the concentric components.

Factory joints are prepared ashore for SUB, while field joints are manufactured for other technologies. The use of prefabricated or pre-moulded joints has brought a significant growth to UGC systems.

The joining of SCC systems is performed by means of a manual process.

#### 2.2.7. Repairability

The repairing of OHL is easier, with visual inspection and direct access to the bare conductors both possible. A preliminary fault location is required for buried technologies, followed by digging to prepare a repair joint. The use of ducts gives the possibility to replace the damaged section by a spare.

The repair of SUB links may require several months and several tens of millions of euros (fault location, raising of cut ends to the water surface, routing of the vessel to the zone, on-site manufacturing of the repair joint).

The repair joint of SCC systems is made more complex by the additional reconstitution of the thermal insulation and associated vacuum loss.

#### 2.2.8. Resilience and Contingency

The possibility to transmit bulk power in the same link may be a drawback in case of a breakdown. The (n-k) rule of parallel circuits per phase is applied to avoid a total power outage. OHL towers often support several circuits and bundles of conductors.

A meshed grid can transfer the load of the failing link to other links, a 3 GW limit often being considered by the transmission system operators. This criterion must be taken into account for SCC systems, whose power rating may exceed this limit. Their intrinsic capability may be then restricted by operating considerations.

#### 2.2.9. Monitoring and Maintenance

OHL are exposed to climatic conditions and corrosion. Frequent visual inspections and tower painting are scheduled.

Very low maintenance is required for UGC and SUB systems, while GIL and SCC have fluids under pressure and associated monitoring and maintenance operations.

#### 2.2.10. Generated Electromagnetic Fields

Electric fields are characteristic of OHL, while magnetic fields are generated by all the technologies under consideration here. It must be noticed that the possible health effects of electromagnetic fields are very different from the static fields generated by HVDC systems [20,21]. Therefore, consistent values for recommendations or regulations are very different for DC and AC (e.g., 400 mT >> 200 µT).

A peak of the magnetic field is observed for UGC above the cable route, but in a very narrow corridor, while a measurable value remains at 100 m for OHL. By design, electromagnetic fields are negligible for GIL and SCC.

#### 2.2.11. General Considerations

Economic considerations are crucial for the selection of the technology. The investment cost (CAPEX) includes the cost of the components, the commissioning tests, but also the delivery, installation on site, and civil engineering. The operating cost (OPEX) includes the cost of monitoring and maintenance, repair, losses. The selection of the technology needs to strike a good balance between CAPEX and OPEX. An attractive CAPEX may lead to an expensive OPEX, due to excessive maintenance operations or equipment replacements. From a technical point, only SCC systems have no electric losses, the remaining technologies exhibit Joules losses depending on the instantaneous current value, which impacts significantly the OPEX. However, the cooling system of a SCC consumes electric power to maintain the operation at cryogenic temperature. In fact, this is the main operating cost of a SCC system. It is difficult to rate the technologies generically, because each project must be assessed separately, accounting for the specific context. It can be noticed that the most expensive projects are those that end up not being realized due to excessive delays or cancelations. Some niche cases can then provide very valuable solutions; for instance, SCC systems can save investments on transformers and substations, due to their lower rated voltage level.

The environmental impact of a transmission system is increasingly considered for the selection of the technology. It can be assessed with a life cycle analysis (LCA) approach. Such studies [22] show for instance that more than 95% of the impact on climate change is caused by operating losses. Apart from the criterion of visual impact, the component manufacturing and the civil engineering (access tracks and concrete foundations of OHL towers, trenching for buried technologies) must be also considered. The compactness of SCC and their narrow trench holds promise for reducing the depletion of raw materials.

These considerations illustrate the complexity involved in the technology selection for a new HVDC power link. In the electricity grid, a combination of the various HVDC technologies is generally used for power transmission along hundreds of kilometers. The different segments using successively one of these technologies are connected in series to build up power links running from remote energy sources to consumption centers in large urban areas.

As discussed above, each HVDC solution has benefits but also limitations according to the specific application. The selection of the best technology for each link segment is strongly dependent on the local installation conditions. For example, the OHL technology is well adapted and accepted in sparsely populated areas, but often rejected in western Europe, in particular in suburban areas. By contrast, given its very small footprint and low environmental impact, the SCC technology is very well adapted to enter or cross large cities or protected areas, but of less interest for crossing large empty areas.

The OHL, UCC, SUB, and GIL technologies have already been installed and proven their added values in electricity grids. Only high-power SCC have not yet been field tested in the real grid. This was the key motivation for the study of HVDC superconducting cable systems in the Best Paths project.

### 2.3. Main Components of a Superconducting Cable System

**Figure 2:**
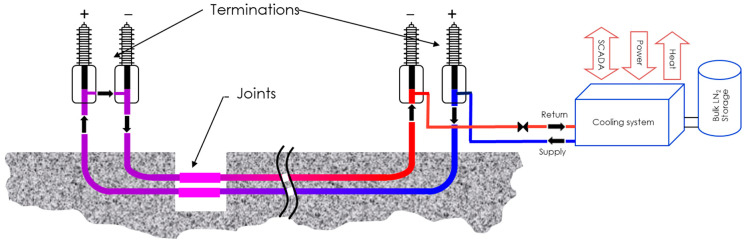
Schematic of a high-voltage direct-current (HVDC) superconducting cable system with a length of approximately 10 km.

Generally, an HVDC superconducting cable system is operating at high voltage with a range of possible currents exceeding 3 kA. Below this current level, the conventional resistive cable technology is more able to meet the grid’s requirements and can be used instead. As seen in Figure 2, the HVDC cable system is bipolar, meaning that the transmission of electricity can take place in both directions.

Essentially, a superconducting cable system consists of the following key components, schematically illustrated in Figure 2:

○ Superconductor;
○ Cryostat (cryogenic envelope), housing the cooling fluid needed to maintain the superconductor temperature;
○ High-voltage electrical insulation;
○ Cryogenic terminations and joints;
○ Adequate cooling devices connected to associated power and fluid supplies for the auxiliary equipment (chiller, pumps, etc.).

The superconducting material is directly wound on a central former that can be practically made with copper cable. This copper core helps to protect the cable during a possible short circuit or fault, where a huge current could suddenly overwhelm the system. To ensure the voltage insulation between the pole and ground, a thick dielectric material is required. This insulation could, for instance, be made out of polyethylene layers and be located outside the cable cryostat. In this case, the insulation operates at room temperature in a so-called “warm dielectric” design [23]. An alternative is to place the high-voltage insulation close to the cable conductor at cryogenic temperature, which constitutes a so-called “cold dielectric” design [24]. The return pole concept is identical.

**Figure 3:**
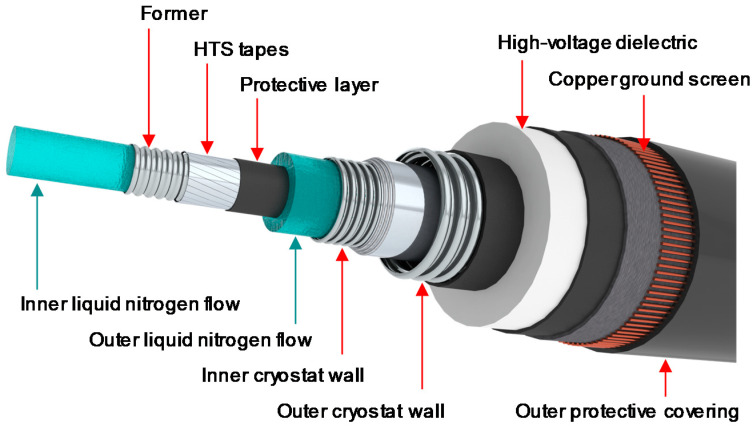
Warm dielectric concept for a high-temperature superconducting HVDC cable.

**Figure 4:**
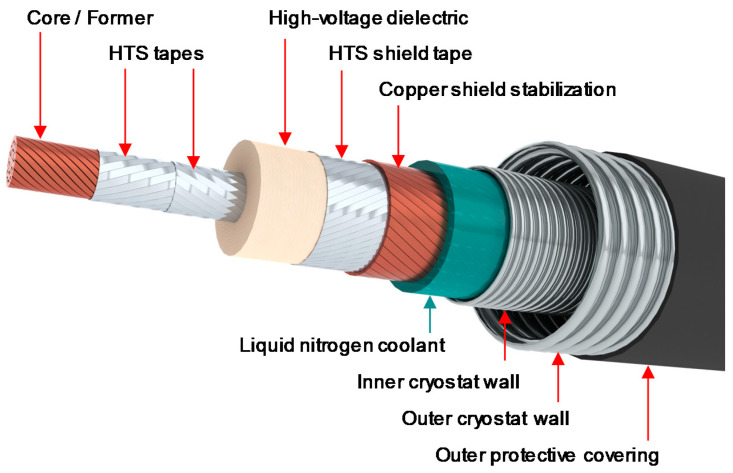
Cold dielectric concept for a high-temperature superconducting HVDC cable.

The cable assembly is placed into a cryogenic envelope with low thermal losses, which maintains the adequate cryogenic operating temperature and prevents thermal exchanges with the surrounding environment. Figure 3 and Figure 4 show the two cable designs for the HVDC cable system. The proposed designs are versatile, allowing for different nominal currents and, therefore, different transmission capacities without an increase in size.

In addition to the cable itself, the system includes two terminations to connect to the electricity grid, some junctions associated with thermal shrinkage management to extend the cable length, and from place to place several cooling systems or refreshing units to recover the operating temperature.

## 3. Dichotomic Decision Optimization for an HVDC Superconducting Link

From the considerations presented in Section 2, it becomes clear that the optimization process for the complex system represented by an HVDC superconducting cable represents a good case study for dichotomic decision making. The various dichotomic possibilities for such a cable system are primarily related to the choice of superconducting material, cooling fluid(s), cryogenic envelope, and electrical insulation, and are all intertwined. They are described and discussed in detail in the following sections.

Before proceeding, a brief summary of the Best Paths superconducting project is provided. The project focused on validating HVDC superconducting links operating at the multi-gigawatt level [13]. In a timeframe of four years (2014–2018), a full-size cable system demonstrator operating at 320 kV and 10 kA was specified, designed, optimized, manufactured, and successfully tested on industrial test platforms. The operating voltage of 320 kV was chosen to facilitate the insertion into the transmission grid, while the current of 10 kA is the maximum amount achievable by converters at present. The testing program was inspired by recommendations and standards for HVDC cables and accessories [14].

Best Paths had an extensive preparatory phase, which encompassed the specification, development and optimization of the main system components. Some of the key considerations elaborated in this preparatory phase follow a dichotomic decision pattern and are incorporated below. Most of the presented tree branches are new and have been explored during the decision-making process. Note that the choices made within Best Paths were partly determined by the scope of the demonstration and the available technologies, as the project aimed for realistic solutions that could be readily implemented.

### 3.1. Superconducting Materials and Cooling Fluids

#### 3.1.1. Superconductor Selection

The selection of the superconducting material has an important impact on the cable design and the choice of the remaining system components, as presented below.

Today three superconducting materials are available in sufficient quantity with high enough performance to be considered for power cables. Their main characteristics and costs are reported in Table 2. The first two are high-temperature superconductors called Bi2223 and YBCO and are both commercially available as tapes. They are cooled using liquid nitrogen (<77 K), a cheap, abundant, and environmentally friendly fluid, which makes them an attractive solution for superconducting grids. However, their costs remain high. Despite an industrialized process based on the powder-in-tube (PIT) technology [25], the Bi2223 tape requires a bulk silver matrix that represents more than 50% of the tape cross section and drives the costs high. For YBCO tapes, the production processes remain costly and complex and result in low yields.

The third material is MgB_2_, available in round wires. They benefit from the high-yield and low-cost PIT process [8], and do not require any expensive raw materials like Bi2223. Consequently, they are available in long lengths and their cost is by comparison low, as indicated in Table 2. However, to be superconducting, this material must be kept below 25 K, a temperature that is not reachable by liquid nitrogen. Other more expensive cooling fluids need to be selected. Additionally, to maintain this cryogenic temperature, sophisticated cooling systems are necessary, which consequently increases the cost and reduces the efficiency. Nevertheless, the technology of such large cooling systems is already fully developed and has demonstrated its reliability and robustness by operating for several years in high-energy applications [27], at even lower temperatures (1.8 K) than the one needed for MgB_2_.

**Figure 5:**
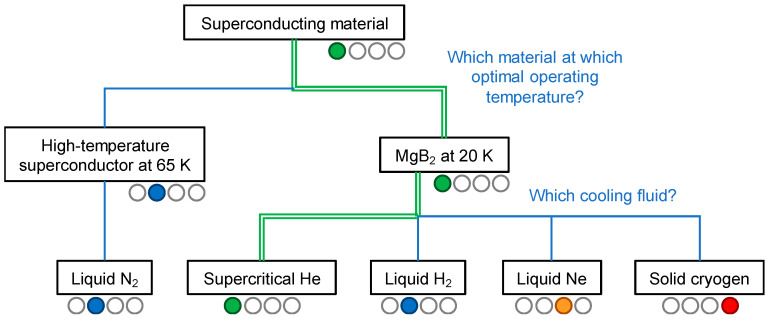
Decision tree on the selection of the superconducting material and associated cooling medium. The color code used here is as follows: green for the selection made in Best Paths, blue for other available alternatives, orange for unsuitable alternatives, and red for alternatives currently no available.

As HVDC superconducting links are foreseen to transmit a high current (>10 kA) across hundreds of kilometers, the use of HTS tapes for such long links was not considered as viable at present. This shall be definitely reconsidered when their cost becomes affordable. Combining the HTS tapes with more expensive cooling fluids such as helium or hydrogen is currently not considered realistic for practical applications, and was therefore not included in the decision tree. Due to its low cost, MgB_2_ was identified as a good candidate, assuming that it is possible to reliably maintain the low operating temperature (<25 K) at an acceptable price. This choice is reflected in the decision tree of Figure 5.

#### 3.1.2. Fluid Selection for the MgB_2_ Superconducting Cable

In a cable system, the temperature of the cooling medium along the link increases between two cooling machines, due to the heat inleak through the cryogenic envelope. To ensure a temperature of 25 K on the MgB_2_ cable, the inlet temperature of the fluid should be set at a lower level, typically at around 15 K. The different options for the cryogenic fluids are illustrated in Figure 5 and discussed below.

Solidified low-cost cryogen fluids such as nitrogen (N_2_) and air were considered but rejected, due to the extremely high number of cooling systems required to be installed along the link, and to the very long cooldown, which is only possible by thermal conduction. For a cable system a circulating fluid is preferred.

The only practical fluids at this temperature are helium (He), hydrogen (H_2_), and neon (Ne). Their physical properties are presented in Table 3. As the operating and investment costs of an HVDC cable system are proportional to the number of cooling machines distributed along the link, it is desirable to reduce their number by selecting the appropriate cooling fluid. The length between two cooling machines increases when the enthalpy span and the density of the fluid increase [28].

Apart from its extremely high cost, Ne solidifies at a temperature of 25 K and evaporates at 31 K, which reduces the operating temperature range of the cable system to the interval 25–30 K. In this range, however, the superconducting properties of MgB_2_ are significantly diminished, nearly vanishing at 30 K. This fluid was thus not considered as viable for an MgB_2_ power cable.

Liquid H_2_ is abundant, and currently foreseen as the energy vector for the future decarbonized world. As a cooling fluid, it offers a suitable operating temperature range of 15–24 K, matching well with the requirements of MgB_2_ wires. Despite a low density, its high specific heat combined with its low viscosity provides sufficient cooling for an MgB_2_ superconducting cable. Even though H_2_ appears the optimal choice, with excellent thermodynamic properties very well suited for MgB_2_ cables, its flammability when coming into contact with oxygen still needs to be carefully studied to become accepted by the public at large. In this regard, the necessary risk management involves the testing and certification of the relevant equipment by authorized bodies. Such studies did not fit with the scope of Best Paths; however, this solution should be seriously considered in future projects.

Supercritical He is relatively rare and expensive when compared to N_2_, but remains affordable relative to Ne [28]. It is easy to handle and poses no flammability risks like H_2_. In supercritical state, it offers a large operating temperature range that can go down to 6 K. However, its low density makes its cooling power lag behind the other fluids under consideration. This can be compensated by an increase of the mass flow circulating through the cable due to its low viscosity. Another advantage of He is that large cryogenic machines and circulation pumps are commercially available and have proven their robustness for more than 30 years of operation [27]. For all these reasons, supercritical He was selected as the cooling medium for the Best Paths project. This choice has an impact on the design and location of the high-voltage insulation, as described in Section 3.4.

### 3.2. Design of the Cryogenic Envelope

The superconducting cable is inserted into a cryogenic envelope that maintains the operating temperature along the full length of the link. Flexible cryogenic lines [26,28] are proposed, as they simplify the cable laying. A cryogenic envelope consists of two concentric corrugated tubes separated by a vacuum chamber to prevent thermal exchange between the “hot” outer wall and the “cold“ inner wall. Superinsulation layers are lapped into the chamber to prevent radiative heating and the gap between the two tubes is maintained by non-thermally conductive spacers distributed along the line. The cryogenic envelopes are designed to be tight to the chosen cooling fluid and to withstand significant pressures of several tens of bar. They are prepared into an appropriate workshop, transported on drums to the installation site, and installed similarly to resistive cables. These cryogenic lines are commercially available and have proven their reliability for more than 50 years. Another solution that could be investigated in the future is to build the cryogenic pipe directly on site, by welding straight segments and then pumping the vacuum chamber in the field. This solution was not studied in the Best Paths project.

Although these cryogenic envelopes have extremely high thermal insulation properties, a limited heat inleak is inevitable. Generally, below 77 K, the insertion of a thermal shield around the cable is considered, as will be discussed in detail in Section 3.3. The thermal shield operates at an intermediate temperature, intercepting the heat flux from the ambient temperature to the cryogenic temperature.

The heat load is dependent on the design of the cryostat. As an example, let us consider an inner tube diameter of 40 mm for the cable conductor, and an inner diameter of 120 mm for the thermal shield. These large dimensions are adequate for long-length links. The average heat inleak of 1 m^2^ of cryogenic envelope and the electrical power required to maintain 1 m of cryogenic envelope at 15 K are presented in Table 4. Two different temperature values for the thermal shield are also compared. Above 100 K, the empirical calculation model used to estimate the heat inleak of the cryogenic envelope is no longer validated by measurements.

As shown in Table 4, the addition of a thermal shield reduces the power consumption of the cable system by more than a factor of 2. When the temperature of the thermal shield increases, the total electrical power needed decreases slightly by 20%. Below 50 K, the thermal shield loses its efficiency and the required power increases sharply. For comparison, the power consumption of a 10 kA resistive cable system built with copper and loaded at 1 A/mm^2^ is 230 W/m. This emphasizes the benefit of using superconducting cables in power grids.

The addition of a thermal shield complicates the power link concept and increases the cost of the overall system. However, this additional cost remains moderate and is quickly compensated by the achieved energy savings, particularly for long HVDC links in operation for several decades. Furthermore, flexible cryogenic lines built with a thermal shield have been developed and are commercially available. For these reasons, the integration of a thermal shield into the cable design was selected for the Best Paths project.

### 3.3. Fluid Selection for the Thermal Shield

**Figure 6:**
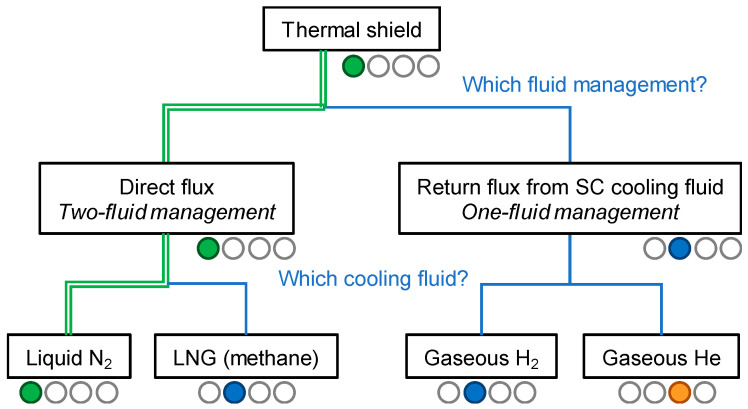
Decision tree on the selection of the cooling medium for the thermal shield. The color code used here is as follows: green for the selection made in Best Paths, blue for other available alternatives, and orange for unsuitable alternatives.

Several cryogenic fluids are possible candidates for a thermal shield between 60 and 100 K. Ideally, they should be cheap, abundant, and either liquid or gaseous. As presented in the tree of Figure 6, two concepts have been envisioned. The first one introduces an additional cryogenic fluid, whereas the second one benefits from the return flux of the cooling medium used for the superconducting cable.

The first option requires the management of an additional fluid. This fluid could be a neutral fluid such as liquid N_2_ or an energy vector, such as liquefied natural gas (LNG). As shown in Table 5, both fluids have similar thermal properties, however using LNG instead of liquid N_2_ shifts the temperature of the shield from 76 K to 105 K.

Furthermore, large industrial liquefaction stations with their associated auxiliaries (pumps, valves) have been in operation for many decades in harsh environments (offshore, tankers), which demonstrates their reliability and robustness. The use of LNG as a thermal shield fluid also offers the possibility to transfer both electrical and chemical energy along the same line. Moreover, a number of distribution gas points could be installed along the link, building a new gas network that will increase the profitability of the system. For a safe use, the association of LNG with the gigawatt-level powers of an electrical transmission cable needs to be carefully validated and certified by authorized bodies.

The second option involves the use of the return flux of the superconductor coolant and would simplify the management and installation of the system. It offers the possibility to operate at a lower temperature than 65 K, where liquid N_2_ solidifies. Naturally, the fluid selection depends on the cooling medium of the superconducting cable, which also limits the design possibilities for the electrical insulation, as discussed in Section 3.4.

Gaseous H_2_ is obtained by the evaporation of liquid H_2_ from the superconducting cable. As presented in Table 5, H_2_ is once again the best fluid with regard to thermal properties. However, as already mentioned, the risk management of its flammability needs additional thorough investigations.

The use of warm gaseous He extracted directly from the end of the superconducting cable channel is also possible. However, this solution has the drawback that it increases the global He inventory of the cable system by at least a factor of 3. While this may be a realistic option for medium-length links, it could significantly impact the cost of the system and possibly the available global He resources for very long links.

While the discussed options are promising and must be deepened, liquid N_2_ was selected as the thermal shield fluid for Best Paths project; this second cryogenic fluid will also contribute to the high-voltage insulation of the cable.

### 3.4. High-Voltage Insulation

Best Paths aimed to demonstrate the feasibility of an HVDC superconducting cable system able to transmit electric powers higher than 3 GW. The 320 kV operating voltage was chosen to facilitate the insertion of the superconducting cable into existing grids, considering the currently deployed grid technologies such as converters, breakers, etc. Consequently, the cable system had to be tested at nearly 600 kV, in order to meet the recommendations for testing HVDC cables from Cigré [30].

This high voltage (HV) level impacts significantly the design, in particular the cable diameter. The thickness of the HV insulation layer is proportional to the testing voltage, and inversely proportional to the voltage breakdown strength of the material and the diameter of the cable conductor. To meet the Cigré testing protocol of a 320 kV class cable insulated by a material with a voltage breakdown strength of 37 kV/mm [14], the insulation layer should have a thickness of at least 27 mm when lapped on a conductor diameter of 32 mm. This insulation layer thus takes significant room in the cross section of the cable and is worth minimizing.

As already mentioned, superconducting cables are designed for electricity transmission at high currents. In order to keep the overall diameter of the cable as small as possible, it is worth reducing the voltage class in future, while increasing the operating current to keep the gigawatt powers [28].

**Figure 7:**
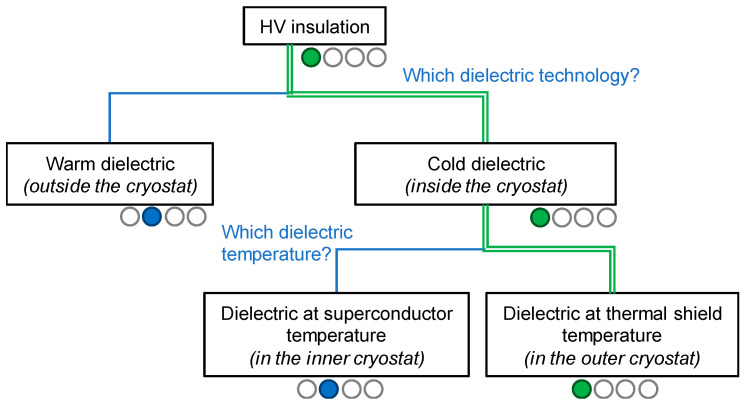
Decision tree for the high voltage (HV) insulation concept of the Best Paths project. Here, green is used for the selection made in Best Paths, and blue for the other available alternatives.

The “warm dielectric” and “cold dielectric” insulation concepts were introduced in Section 2.3. The “warm dielectric” is an attractive solution, proven for several decades in the electricity grids. However, for long-length HVDC superconducting cables, the outer cryostat of the thermal shield needs to be larger than 150 mm to have sufficient cooling capability. Extrusion lines able to sheath HV cables with such dimensions are uncommon in the cable industry. Consequently, this solution was not considered for Best Paths, as illustrated in the decision tree of Figure 7.

In the “cold dielectric” design, the employed material needs to be sheet-structured to avoid cracks in the HV insulation layer, due to differential shrinkage during cooldown [24]. Appropriate materials include lapped paper immersed in a cryogenic fluid with good dielectric properties, or lapped polymer tapes such as polyimide or polypropylene. In the first case, the dielectric material is mainly the cryogenic fluid. The paper provides a porous space between the elements under HV and ground. The second case benefits from the high dielectric properties of the polymer tapes themselves. This kind of insulation is selected when the element under HV is immersed in a poor dielectric medium, such as a gas or vacuum, and is preferred for low- to medium-voltage systems. The polymer material and the tape preparation can be really expensive especially for high voltages, where a thick layer of insulation is required. Both solutions have proven their reliability in many electrotechnical systems (magnets, transformers, motors, etc.), fault current limiters, or superconducting cable projects.

As a thermal shield is envisioned around the cable for Best Paths, there are two possible locations in the “cold dielectric” design. The first one involves lapping the insulation layer directly onto the cable conductor and placing the arrangement into the inner cryogenic envelope. The material used will depend then on the cooling fluid. For liquid H_2_, paper can be selected as the insulation layer, while for gaseous He polyimide tapes are recommended due to its poor breakdown voltage. Furthermore, the 10 kA conductor composed of MgB_2_ wires has an outer diameter of 10 mm. For a material with a dielectric breakdown of 37 kV/mm, the insulation thickness for a 320 kV class system is estimated at about 110 mm, due to local electric field strength. This solution is not realistic. However, decreasing the voltage to a 100 kV class, for example, reduces the insulation thickness down to 8.5 mm, which becomes acceptable. As the Best Paths system is specified to operate at 320 kV, the solution of a cold dielectric located in the inner cryostat was not chosen for the project (see Figure 7).

The second possible location for the HV insulation sheath is directly on the external wall of the inner cryogenic envelope. This assembly is thus inserted into the thermal shield conduit, and the paper insulation is immersed in the cryogen for the shield. In addition to its efficient cooling properties, liquid N_2_ also has good dielectric strength properties [31]. As the diameter of the external wall of the inner cryostat is about 50 mm, the insulation thickness is 23 mm at the required dielectric breakdown, which represents a reasonable value.

**Figure 8:**
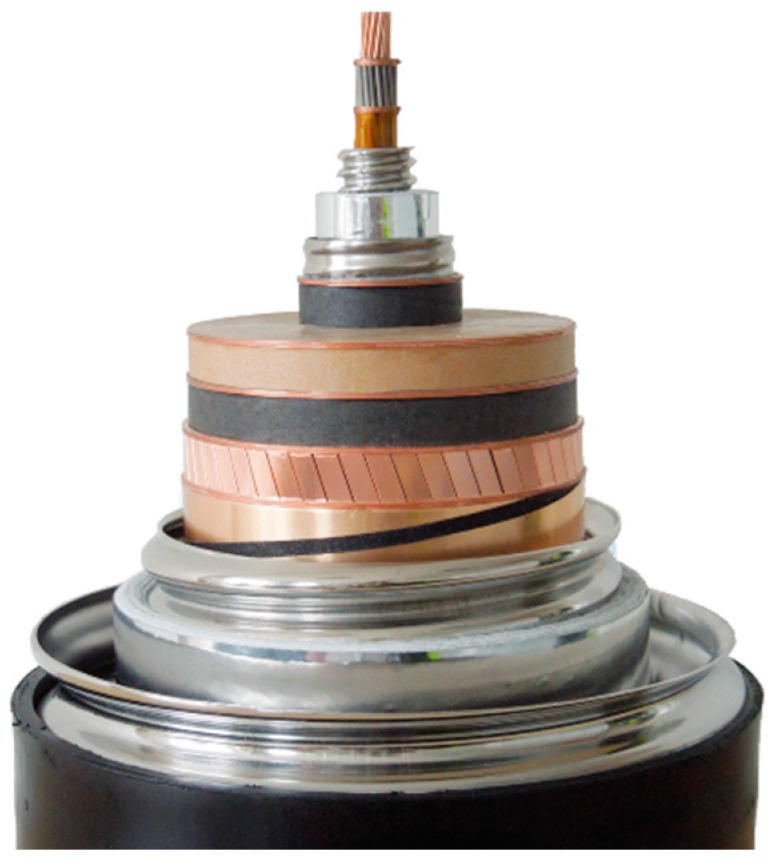
Final demonstrator design successfully tested in Best Paths.

In conclusion, the HV insulation design selected for Best Paths is “cold dielectric“, located in the thermal shield and immersed in liquid N_2_. A photograph of the cable assembly that was manufactured and tested in the project is shown in Figure 8.

### 3.5. Protection of the Cable System

The protection of the system is developed according to the technology and performance of converters and circuit breakers. Fault scenarios in the grid must be studied to make sure that the cable withstands accidental conditions, during which the fault current may reach several tens of kA until the circuit is opened.

For resistive cables, the over-current fault management is aimed at avoiding damages to the HV insulation. For superconducting cables, design efforts are directed at managing the cable during the fault and the ensuing transition of the cable from the superconducting state to the normal resistive state (so-called quench). Two distinct approaches can be used for this purpose [32]:

- The “fault tolerant” conductor design withstands the quench, with an over-current during the fault but no damage to the conductor. This is due to the copper core of the cable (see Figure 4), which acts as a low-resistance electrical shunt protecting the superconducting wires and transporting the current in excess of the superconducting critical current. The cable must be disconnected from the grid and can be energized again after the recovery of the operating temperature.
- The “fault transparent” conductor design is enabled by the high current and good thermal stability of the MgB_2_ superconductor, which come at an affordable cost. In such a design, a conductor consisting of multiple high-current MgB_2_ strands can withstand the fault current without a quench.

A fault-tolerant design was chosen for Best Paths, and the behavior of the cable conductor was investigated for an over-current of 35 kA lasting 100 ms. This level of fault current is a representative value required by transmission system operators for a long HVDC cable link. The diameter of the copper core can be chosen according to the level and the duration of the fault current. To determine the optimal diameter, it is essential to evaluate the maximum temperature reached, so as to avoid any damage by overheating. To this end, a multi-physics model has been developed taking into consideration the thermal and electrical properties of the temperature-dependent materials. For the chosen Best Paths design, the simulations show that the temperature does not exceed 90 K during and after the investigated fault [32].

In general, the value of fault current should be calculated considering the real grid architecture, in particular, the power available and the impedance of all the circuits connected together.

Apart from the engineering and practical aspects discussed so far, the requirements for an HVDC superconducting cable should also consider other aspects that impact the technology selection. For long links, the selected cable design should be manufacturable on a large scale over long lengths, with a minimal number of joints. During the installation phases, the cable should be transportable to the different sites along the link, and efficiently laid and buried into the ground. The duration and impact of civil engineering should be kept as limited as possible, in order to improve the obtaining of administrative authorizations. All required rules and regulations should be followed, while also addressing the concerns of the affected local actors. Once in operation, it is crucial that the cable system be robust, reliable, and maintainable.

## 4. Conclusions and Future Work

This article presents the application of a dichotomic decision process to achieve an optimized design for an HVDC superconducting link. This complex process is exemplified using the European project Best Paths, in which the first 3-gigawatt-class HVDC superconducting cable system was manufactured and successfully tested.

A number of different conceptual and technological options for the practical realization of high-power superconducting links are described in detail, even if they were not feasible within the scope of Best Paths. To begin with, superconducting links are placed into the landscape of available HVDC technologies in a detailed overview. Afterwards, the main part of the article focusses on the dichotomic possibilities related to some of the key components of the cable system, such as the superconductor, cooling fluid, cryogenic envelope, thermal shield, and high-voltage electrical insulation. The associated analysis should provide a good basis for designing a variety of configurations for superconducting links.

Superconducting links represent a technically realistic solution for bulk power transmission and could contribute to the necessary decarbonization of our society by reinforcing and increasing the efficiency of electricity grids with very limited environmental impact. Moreover, a whole panoply of concepts and technologies exist that can still enhance their efficiency and carbon emission footprint. Beyond electricity transmission, the use of superconducting cables also opens up the unique and promising possibility of simultaneous transport of two energy carriers, by combining both hydrogen and electricity.

## Figures and Tables

**Table 1:** Rating of available technologies for high-voltage direct-current (HVDC) transmission, according to various design criteria.

Criteria	OHL	SUB	UGC	GIL	SCC	Item
Visual impact	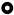 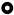 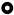 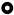	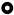	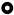	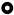 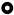	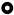	2.2.1
Infrastructure footprint and right-of-way	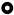 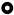 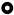 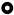	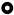	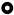 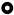	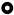 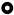	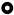	2.2.2
Sensitivity to climatic hazards	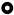 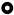 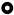 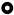	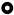	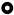	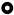	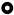	2.2.3
Sensitivity to ambient temperature	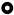 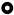 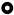	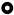	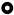 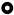 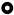 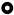	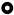 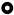 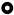	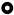	2.2.4
Sensitivity to rocky environment	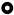	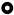 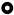 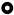	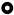 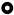 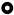	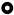 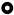 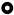	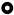 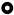 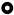	2.2.5
Ease of making joints	★★ ★★	★★	★★ ★	★★	★	2.2.6
Repairability	★★ ★★	0	★★	★★	★	2.2.7
Resilience and contingency	★★ ★★	★	★★	★★	★	2.2.8
Monitoring and maintenance	★	★★ ★★	★★ ★★	★★	★★	2.2.9
Generated electromagnetic fields (EMF)	★	★★ ★	★★	★★ ★★	★★ ★★	2.2.10
Established grid technology	★★ ★★	★★ ★	★★ ★	★★	★	

**Table 2:** Main characteristics of superconducting materials for power cables, see for instance [26].

	Shape	Dimensions		Performance of Commercial Tapes and Wires		
Bi2223	Laminated PIT * tapes	Width 4.5 mm	Thickness 0.3–0.5 mm	Ic @70 K, 0.5 T 350–400 A.cm^−1^	Length < 1500 m	80–120 €/kA/m
YBCO	Laminated thin film coated tapes	Width 4–12 mm		Ic @70 K, 0.5 T 500–800 A.cm^−1^	Length < 500 m	
MgB_2_	Cylindrical PIT * wires	Ø 0.8–1.5 mm		Ic @20 K, 1T 400 A/mm^2^	Length < 3000 m	3–5 €/kA/m

* PIT: Powder-inTube technology.

**Table 3:** Thermodynamic characteristics of possible cooling fluids for MgB_2_ [29].

	Liquid N_2_	He *	Liquid H_2_	Liquid Ne
	Abundant and safe	Not rare and safe	Abundant but flammable	Very rare and safe
T solidification (K)	65	N/A	15	24.6
T boiling (K) at 3 bar	87	5	24	31
Cp (J/kg.K) _(P, T)_	2000_(70K)_	5300_(20K)_	9400_(20K)_	1800_(27K)_
ρ (kg/m^3^)	830_(70K)_	5.7_(3bar,20K)_–9.8_(20bar,20K)_	72_(20K)_	1212
Viscosity (µPa.s)	225_(70K)_	4_(20K)_	15_(20K)_	120_(27K)_

* He is supercritical in this range of pressures and temperatures and its density is pressure-dependent.

**Table 4:** Comparison of the electrical powers required to maintain the cryogens at 15 K in flexible cryogenic envelopes.

	No Thermal Shield	With Thermal Shield	
	SCC at 15 K	Shield at 60 K SCC at 15 K	Shield at 100 K SCC at 15 K
Heat inleak at the cold wall (W/m^2^)	7.3	Shield 4.5 Cable 0.7	Shield 4.5 Cable 1.3
Electrical power to maintain the shield temperature (*D_in_ =* 120 mm) (W_elec_/m)	N/A	22	12
Electrical power to maintain the SCC at 15 K (*D_in_ =* 40 mm) (W_elec_/m)	57	5	10
Total electrical power (W_elec_/m)	57	27	22

**Table 5:** Average characteristics of the possible fluids for the thermal shield [29].

	Direct Flux		Return Flux	
	Liquid N_2_	LNG	Gaseous He	Gaseous H_2_
	Abundant and safe	Abundant but flammable	Common and safe	Abundant but flammable
T solidification (K)	65	91	N/A	N/A
T boiling (K) at 3 bar	87	120	N/A	N/A
Cp (J/kg·K)_(T)_	2030_(76K)_	3400_(105K)_	5200_(60K)_	10,700 _(60K)_
ρ (kg/m^3^)	812_(76K)_	430_(105K)_	2.4_(3bar)_	1.25_(3bar)_
